# Supplementary material for: Beyond scores: A machine learning approach to comparing educational system effectiveness
Source: PLoS One. 2023 Oct 26;18(10):e0289260. doi: 10.1371/journal.pone.0289260 (PMC10602239; doi:10.1371/journal.pone.0289260)
Supplement: S1 Appendix — (DOCX) [file pone.0289260.s001.docx]

Appendix A

| ID | Variable | Mean | S.D. | Min | Max | Source | Type |
| --- | --- | --- | --- | --- | --- | --- | --- |
| **1** | *Computer lab* | 0.89 | 0.31 | 0.00 | 1.00 | School | Binary |
| **2** | *Science lab* | 0.55 | 0.50 | 0.00 | 1.00 | School | Binary |
| **3** | *Special attendance room* | 0.22 | 0.41 | 0.00 | 1.00 | School | Binary |
| **4** | *School library* | 0.74 | 0.44 | 0.00 | 1.00 | School | Binary |
| **5** | *Reading room* | 0.37 | 0.48 | 0.00 | 1.00 | School | Binary |
| **6** | *W.C.* | 0.97 | 0.17 | 0.00 | 1.00 | School | Binary |
| **7** | *Disabled W.C.* | 0.54 | 0.50 | 0.00 | 1.00 | School | Binary |
| **8** | *Classrooms* | 14.96 | 8.18 | 4.00 | 42.00 | School | Numeric |
| **9** | *Television* | 0.96 | 0.20 | 0.00 | 1.00 | School | Binary |
| **10** | *DVD player* | 0.88 | 0.32 | 0.00 | 1.00 | School | Binary |
| **11** | *Copy machine* | 0.71 | 0.45 | 0.00 | 1.00 | School | Binary |
| **12** | *Printer* | 0.93 | 0.26 | 0.00 | 1.00 | School | Binary |
| **13** | *Student Computer* | 21.48 | 19.86 | 0.00 | 102.00 | School | Numeric |
| **14** | *Fast Internet* | 0.87 | 0.33 | 0.00 | 1.00 | School | Binary |
| **15** | *Number of employees* | 65.09 | 36.20 | 12.00 | 175.00 | School | Numeric |
| **16** | *School lunch* | 0.74 | 0.44 | 0.00 | 1.00 | School | Binary |
| **17** | *Faculty room* | 0.97 | 0.18 | 0.00 | 1.00 | School | Binary |
| **18** | *School Kitchen* | 0.91 | 0.28 | 0.00 | 1.00 | School | Binary |
| **19** | *Satellite Dish* | 0.48 | 0.50 | 0.00 | 1.00 | School | Binary |
| **20** | *Sports court* | 0.89 | 0.32 | 0.00 | 1.00 | School | Binary |
| **21** | *Complementary activity* | 0.25 | 0.43 | 0.00 | 1.00 | School | Binary |
| **22** | *Father’s Education* | 2.00 | 0.79 | 0.65 | 3.77 | Student | Numeric |
| **23** | *Mother’s Education* | 2.41 | 0.73 | 1.11 | 3.97 | Student | Numeric |
| **24** | *Faculty education* | 0.14 | 0.08 | 0.02 | 0.34 | Teacher | Numeric |
| **25** | *Income (USD per capita)** | 418.63 | 282.42 | 88.75 | 1190.17 | Student | Numeric |
| **26** | *White students (fraction)* | 0.47 | 0.26 | 0.05 | 0.95 | Student** | Numeric |
| **27** | *Black students (fraction)* | 0.09 | 0.08 | 0.00 | 0.33 | Student** | Numeric |
| **28** | *Brown students (fraction)* | 0.38 | 0.22 | 0.00 | 0.82 | Student** | Numeric |
| **29** | *Yellow students (fraction)* | 0.02 | 0.03 | 0.00 | 0.11 | Student** | Numeric |
| **30** | *Indigenous students (fraction)* | 0.00 | 0.01 | 0.00 | 0.05 | Student** | Numeric |
| **31** | *Student´s gender* |  |  |  |  | Student** | Numeric |
| **32** | *Enrollments* | 29.88 | 8.09 | 14.00 | 48.00 | School | Numeric |
| **33** | *Natural Science faculty (fraction)* | 0.16 | 0.08 | 0.03 | 0.33 | Teacher | Numeric |
| **34** | *Humanities faculty (fraction)* | 0.27 | 0.05 | 0.17 | 0.38 | Teacher | Numeric |
| **35** | *Languages faculty (fraction)* | 0.39 | 0.06 | 0.26 | 0.50 | Teacher | Numeric |
| **36** | *Math faculty (fraction)* | 0.18 | 0.05 | 0.07 | 0.28 | Teacher | Numeric |
| **37** | *Faculty jobs* | 1.38 | 0.32 | 1.00 | 2.36 | Teacher | Numeric |
| **38** | *Pedagogical Training* | 0.90 | 0.11 | 0.57 | 1.00 | Teacher | Numeric |
| **39** | *Faculty adequate training* | 0.68 | 0.10 | 0.47 | 0.86 | Teacher | Numeric |
| **40** | *Student’s age* | 17.55 | 0.30 | 17.08 | 18.33 | Student | Numeric |
| **41** | *Faculty work overload* | 0.85 | 0.17 | 0.47 | 1.00 | Teacher | Numeric |

* Converted from Brazilian Real to United States Dollar using the average rate in the period (2.67)

** Students variables grouped in the non-actionable features
