# Supplementary material for: Beyond scores: A machine learning approach to comparing educational system effectiveness
Source: PLoS One. 2023 Oct 26;18(10):e0289260. doi: 10.1371/journal.pone.0289260 (PMC10602239; doi:10.1371/journal.pone.0289260)
Supplement: Attachment — Submitted filename: Response to Reviewers.docx [file pone.0289260.s002.docx]

**Response to Reviewers**

**R1Q1** - **I suggest that the authors standardize the tables in the manuscript, as well as increase the resolution of the figures to improve the readability of the work. In addition, the figure 5 is with the labels of the regions in Portuguese, unlike what happens in the other figures.**

*R. Thanks for the comment. The tables were standardized under a simple layout, and the figures are present in 400 DPI resolution. Also, the Figure 5 labels were updated.*

**R1Q2** - **It is unclear which fractions of the dataset the authors used for training/testing, as well as whether a sampling strategy was used to ensure a good fit of the models. This is important to facilitate the reproducibility of the results presented in the work. Therefore, I suggest that the authors clarify this in the text.**

*R. Thanks for this comment. The information about the fraction used to train and test the algorithm is explained in the second paragraph of subsection 4.5. To make it clearer, we have updated this paragraph*.

**R1Q3** - **In section "4.5 Modeling and evaluation," the authors consider the use of three classifiers: *logistic regression*, *random forest* and *adaboosting*. Did the authors adjust the hyperparameters for these models? It would be important to make clear which hyperparameters were varied or which values were set if they were kept fixed.**

*R. Thanks for this comment. This information is already presented in the last sentence of the last paragraph in section 4.5. We have used the default parametrization. However, we have updated this paragraph to justify that.*
